# Salmon subsidies predict territory size and habitat selection of an avian insectivore

**DOI:** 10.1371/journal.pone.0254314

**Published:** 2021-07-08

**Authors:** Kirsten A. Wilcox, Marlene A. Wagner, John D. Reynolds

**Affiliations:** Department of Biological Sciences, Earth to Ocean Research Group, Simon Fraser University, Burnaby, British Columbia, Canada; University of Sydney, AUSTRALIA

## Abstract

The annual migration and spawning event of Pacific salmon (*Oncorhynchus* spp.) can lead to cross-boundary delivery of marine-derived nutrients from their carcasses into adjacent terrestrial ecosystems. The densities of some passerine species, including Pacific wrens (*Troglodytes pacificus*), have been shown to be positively correlated with salmon abundance along streams in Alaska and British Columbia, but mechanisms maintaining these densities remain poorly understood. Riparian areas near salmon streams could provide higher quality habitat for birds through greater food availability and more suitable vegetation structure for foraging and breeding, resulting in wrens maintaining smaller territories. We examined relationships between salmon biomass and Pacific wren territory size, competition, and habitat selection along 11 streams on the coast of British Columbia, Canada. We show that male wren densities increase and territory sizes decrease as salmon-spawning biomass increases. Higher densities result in higher rates of competition as male wrens countersing more frequently to defend their territories along streams with more salmon. Wrens were also more selective of the habitats they defended along streams with higher salmon biomass; they were 68% less likely to select low-quality habitat on streams with salmon compared with 46% less likely at streams without salmon. This suggests a potential trade-off between available high-quality habitat and the cost of competition that structures habitat selection. Thus, the marine-nutrient subsidies provided by salmon carcasses to forests lead to higher densities of wrens while shifting the economics of territorial defence toward smaller territories being defended more vigorously in higher quality habitats.

## Introduction

Many seemingly disparate ecosystems are linked by the movement of species and nutrients across habitat boundaries [1]. Predictable pulses of subsidies across ecotones can have a wide range of ecological impacts, from increasing local densities of consumers [2,3] to longer-term, comprehensive effects on recipient ecosystem productivity or community structure and stability [4,5]. Nutrient subsidies enter recipient communities at low trophic levels, but can propagate through multiple trophic levels [4,6,7]. These subsidies can increase local densities of direct consumers, leading to widespread consequences for food web dynamics [8,9]. For example, a study of the effect of a single input of nitrogen fertilization showed that a short resource pulse can have lasting effects on abundance of grasses, but also increase the abundance of herbivorous insects and their arachnid predators for up to three years [10]. Increasing densities of secondary consumers has implications for structuring community dynamics through top-down effects or intraspecific competition [11].

Along the North Pacific Rim, the annual migration and subsequent spawning of anadromous salmon into freshwater streams represents one of the most striking examples of a predictable pulse of nutrient subsidies largely assimilated at sea and transferred to freshwater and terrestrial systems. Nitrogen from carcasses of adult salmon can impact the recipient community directly as food and nutrients for consumers, or indirectly through various bottom-up interactions. Salmon-derived nutrients are exported to terrestrial systems through passive deposition, flooding, or transport by bears, wolves, and birds [12–16]. Salmon carcases are colonized by terrestrial invertebrates and this subsidy has been shown to increase invertebrate biomass the following spring [8,17]. Salmon carcasses can also influence growth and community composition of riparian plants through the release of carbon and nitrogen [18,19] which sustain different herbivorous invertebrate communities [20].

Marine-derived nitrogen from salmon create bottom-up trophic cascades, cumulating in higher densities of riparian passerine birds along salmon streams [21–24]. Nitrogen subsidies from salmon carcasses may increase habitat quality for passerines through greater food availability and potentially better habitat structure that enhances foraging opportunities and nest site availability [25–27]. For example, along salmon streams there is higher invertebrate prey availability [17] and the riparian plant community composition is more salmonberry-dominated (*Rubus spectabilis*) [18], which produce fruit and have been shown to support higher insect biomass than conifers [20]. There is also evidence of an indirect trophic link between salmon subsidies and Pacific wrens, as isotopic shifts in wren tissues reflect the availability of salmon in invertebrate diets [28].

Many studies have examined the indirect effects of subsidies on recipient communities through increases in consumer abundances, biomass, or densities [29]. However, there is less of an understanding of the behavioural processes underpinning these outcomes as densities of consumers are not always correlated with higher resource availability [30,31]. In this study, we attempt to elucidate some of the mechanisms maintaining higher densities of birds through indirect effects of marine-derived salmon subsidies. We examine how individuals may balance the trade-off between access to high-quality resources and competition. We quantify the consequences of competition by measuring habitat use, individual quality or body condition, and local densities [32–34]. As habitat selection is an important component of these processes, territorial species are particularly well suited to understand the mechanisms maintaining higher densities of passerines along salmon streams.

Indirect effects of salmon-derived nutrients on territoriality can be studied through the lens of food-value theory [35,36], which predicts an inverse relationship between food availability and territory size as territories scale to support the energy necessary to live [37]. However, territory size can also be constrained by the trade-off between defending against conspecifics and habitat quality [35]. There may be more pressure to defend high-quality habitat when competitor density is increased, requiring the allocation of more energy and time to maintain a territory. Marine-derived nutrients have been shown to positively influence many indicators of high-quality habitat for insectivorous songbirds including plant community composition and invertebrate availability [18,38]. Therefore, the trade-offs between competition and resource use mediated through nutrient subsidies, can determine territory size [35].

We studied the Pacific wren (*Troglodytes pacificus*) to test for mechanisms that facilitate marine-derived salmon subsidies into higher densities of breeding birds. Pacific Wrens are resident territorial insectivores that range along the west coast of North America from Alaska to California [39]. Male Pacific Wrens establish relatively small breeding territories in the early spring and defend them throughout the spring and summer by countersinging with their neighbours [26]. Their small territories and year-round close association with riparian areas along streams make them an effective model organism to study the localized effects of salmon nutrient subsidies on habitat use and competition.

The objectives of this study were to determine if nutrient subsidies provided by spawning salmon are correlated with the territorial decisions influencing habitat selection, defence, and territory size, leading to higher wren densities. First, we tested the prediction that streams with a higher input of marine-derived nutrients through higher spawning salmon biomass would have higher wren densities with smaller territory sizes. Second, we predicted that competition with conspecifics would be more intense along streams with high densities of salmon, shown by rates of countersinging. Third, we tested whether increased pressure from conspecifics as a function of salmon abundance would lead to wrens selecting only higher quality habitat to defend. Finally, we tested the prediction that wren body condition would be higher for wrens with smaller territories across salmon streams. Thus, our study tests the overall prediction that nutrients derived from salmon would lead to higher densities of wrens while mediating trade-offs between resource availability and competition.

## Methods

### Study sites

Research was conducted under Simon Fraser University Animal Care Protocol #1044B-12 and #1079B-13 and bird-banding permit 10759 AW issued by the Canadian Bird Banding Office. Our study was conducted from April to July in 2015 and 2016 on the Central Coast of British Columbia, Canada, within the traditional territory of the Heiltsuk First Nation (vicinity of 52.1605° N, 128.1456° W). The study sites were located along streams on coastal islands and mainland inlets within the Coastal Western Hemlock Biogeoclimatic Zone, which is characterized by nutrient-poor soils and high annual precipitation of 3000–4000 mm⋅year^-1^ [40]. Forest cover is dominated by western hemlock (*Tsuga heterophylla*), western redcedar (*Thuja plicata*), Sitka spruce (*Picea sitchensis*), amabilis fir (*Abies amabilis*), and red alder (*Alnus rubra*). The latter is the only large deciduous tree found at our sites. Riparian plant understory communities are composed of shrubs including salmonberry (*Rubus spectabilis*), stink currant (*Ribes bracteosum*), blueberries (*Vaccinium ssp*.), false azalea (*Menziesia ferruginea*) and salal (*Gaultheria shallon*). Most of our sites were selectively logged for spruce in the 1940s, however there was minimal evidence of modern anthropogenic disturbances.

We conducted bird and vegetation surveys at 11 stream-side plots, five of which were surveyed in 2015 and six surveyed in 2016. Stream sites were chosen to represent a wide range of salmon (0 to 122,454 fish; S1 Table). We established a nine-hectare forest plot beginning at the mouth of the stream and extending 375 m upstream and 125 m upland on either side of the stream. Standard protocol recommends plot sizes of at least 8 hectares and 4–10 repeated visits for effective spot-mapping of one species [41]. We chose this plot shape and size as Pacific Wrens are more abundant in riparian forests in close proximity to streambanks [29] and in coastal rainforests of British Columbia, previously cited wren territories ranged from 0.68 to 1.46 ha [42]. The streams were generally similar in width, ranging from 5 to 22 m wetted width. Stream width was not included in the size of each terrestrial plot as wrens easily flew from one side to the other and stream width did not correlate with salmon biomass (S1 Fig). We flagged the plots at 50 m by 25 m intervals, creating points to facilitate data collection and to geolocate observations (S2 Fig). We determined the Universal Transverse Mercator (UTM) coordinates for all points using hand-held GPS units.

### Salmon biomass

Salmon counts on each stream were done by collaborative surveys conducted by our research group and from data shared by the Department of Fisheries and Oceans and the Heiltsuk Integrated Resource Management Department. Trained observers walked the streams and counted all live and dead salmon observed along the total length of the stream or until no salmon were seen for 200 m. Streams were surveyed three times over the course of the salmon spawning season from early September to late October to determine spawning salmon abundance using the area-under-the-curve estimation method [43]. When streams had very low salmon abundance or we were constrained by weather to only one or two counts per stream, we used peak counts of live plus dead to estimate salmon abundance. Both methods result in the same mean estimates of run-size [18].

We used total salmon biomass per stream as the metric of marine-derived nutrient subsidy, as all salmon, regardless of stream length would pass through the surveyed area near the estuary of each stream. Additionally, salmon biomass, as opposed to salmon density, has been found to better explain variation in Pacific Wren abundances along the same streams [24]. Streams were heavily dominated by pink (*Oncorhynchus gorbuscha*) and chum (*O*. *keta*) salmon (>99% of fish). Therefore, biomass was calculated as the total pink and chum run size at each stream multiplied by their respective average weights (1.2 kg for pink and 3.5 kg for chum) [18]. We calculated a three-year mean of salmon biomass for each stream for the years before we did our bird surveys to account for longer-term effects of salmon. The streams ranged from having no salmon to an average of 46,609 kg spawning salmon annually.

### Territory mapping and densities

Early in the breeding season, from April to early June, we colour-banded male wrens by catching them in mist nets with targeted playback of their song to aid in individual identification of territorial behaviour. To determine territory size we used spot-mapping, a well-established method for determining habitat use of breeding birds [41,44,45]. We initiated spot-mapping one hour after sunrise each day and continued for 1.5 hours. Observers moved through the plot along the geolocated points at a consistent walking pace, recording the locations of singing, countersinging, calling, and visual observations of male wrens against known plot locations. Each stream was visited eight times each year, from late April until July, in rotating order (one site per day) and for each visit, we began spot mapping at either a different corner of the plot, or by moving in a different direction to ensure adequate coverage throughout the morning chorus.

**Fig 1:**
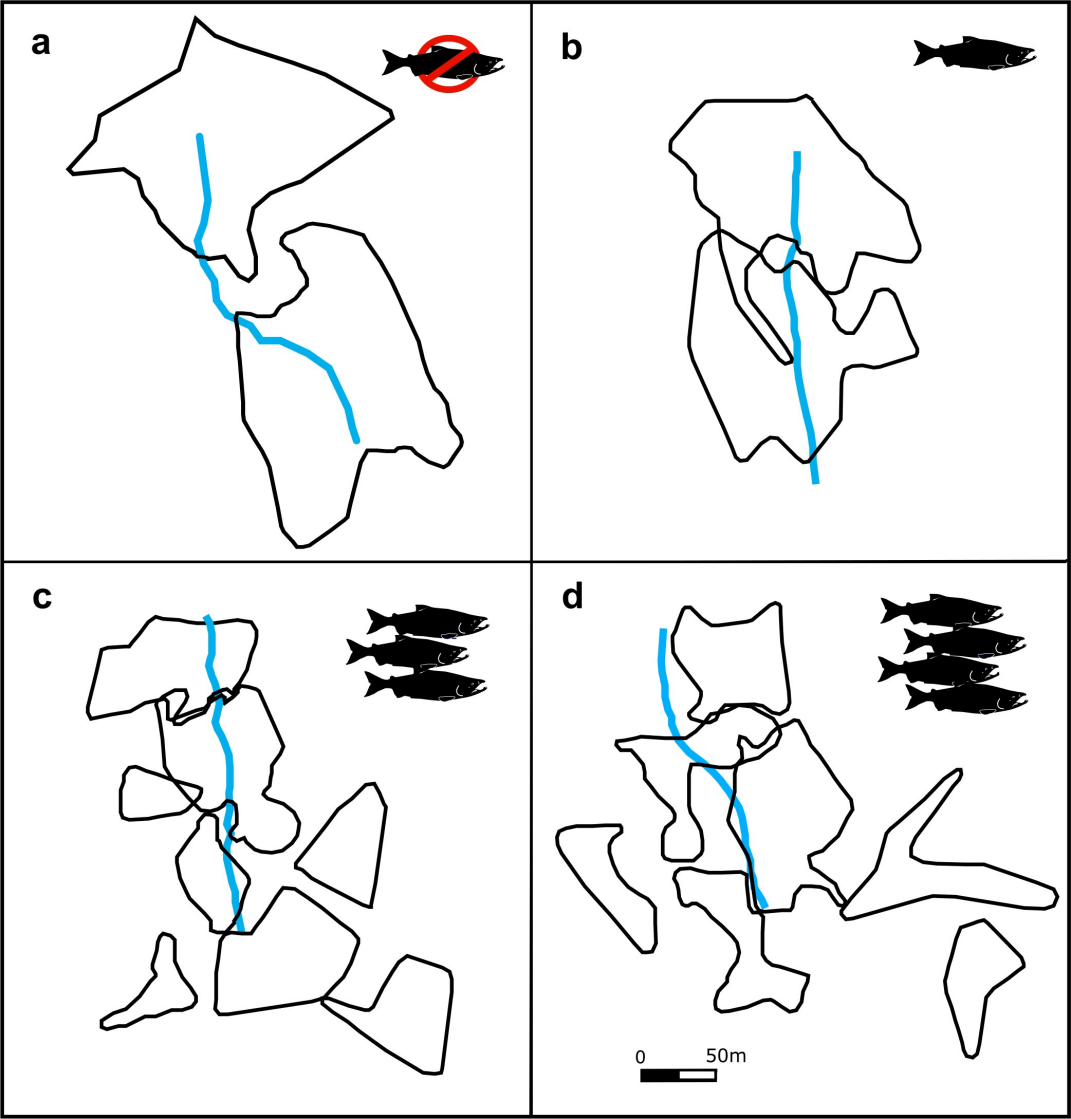
Representation of the territories of male Pacific wrens at different streams with different numbers of spawning salmon (A-D). Each polygon represents an individual wren territory along a stream: (a) Ripley with no salmon, (b) Fancy Right with ~2,500 kg, (c) Fannie Left with 32,000 kg, and (d) Clatse with 47,000 kg average annual spawning pink and chum salmon biomass. Blue lines represent the respective streams and the salmon drawing represents amount of salmon biomass.

Territories were delineated in ArcGIS (Version 10.1) by geo-referencing each song or visual observation against known site points and then overlaying each visit to create an overall pattern, or cluster, of territorial observations throughout the season (Fig 1). Territory boundaries were mapped according to countersinging locations between male wrens. Countersinging occurs when birds sing in response to their neighbour’s song by matching or overlaying songs [46]. Pacific wrens will often countersing at the edges of defended habitat, allowing us to mark the outer borders of territories.

We measured territory size using 95% minimum convex polygons, with the adehabitat package in R [47], using 95% of all singing observations for each wren. Territories that had more than half of their observations outside the surveyed plot area were excluded from the study as their size may have been underestimated. Similarly, male wrens that were detected in fewer than three survey visits or that had fewer than three total observations throughout the breeding season were excluded from the analysis as we categorized them as floaters unable to establish a breeding territory.

To calculate absolute male wren densities at each stream we determined the proportion of each male’s territory within the surveyed stream-site, summed the proportions of the territories in the surveyed area, and divided the sum by the total surveyed stream-site area. This gave us male wren densities as number of territorial males per hectare.

### Countersinging rate

We first determined the number of countersinging bouts attributed to an individual bird in order to determine countersinging rate per male as a metric of competition. Observations were categorized as countersinging if observers heard two male wrens singing in response to each other by either alternating or overlaying songs. We tallied the number of observations of countersinging for each individual bird during our spot-mapping surveys and standardized it by the total number of hours surveyed. Similarly, we calculated the singing rate of each territorial male wren as the total number of singing observations that occurred within a territory, standardized by survey effort (total survey hours at each stream over the season). These methods have been used previously to measure countersinging rates [48]. We then divided the number of countersinging bouts by the total number of singing observations recorded throughout the survey to calculate the proportional rate of countersinging for each individual male wren.

### Vegetation surveys

To characterize habitat quality and wren habitat selection, we surveyed vegetation plots at each geolocated stream-site point (50 m x 25 m) for a total of 56 plots per stream using a modified BBIRD protocol [49]. To measure understory characteristics, percent cover was recorded for all shrub and immature tree species (<2.5 cm diameter at 10 cm or <5 m height) and summed across three different height classes (0–50 cm, 50–200 cm, 200–500 cm) within a 5-m radius. Percent cover was visually estimated by trained observers using a datasheet to draw out cover estimates of each species on circles divided into four quandrants (S2 Fig) [49]. We focused on vegetation below five meters in height because our field observations indicated that this was where wrens spent most of their time. Within the 5-m radius vegetation plot we also measured percent cover of logs and stumps and calculated Shannon’s Diversity Index of tree and shrub species. To calculate the habitat characteristics for each territory, we averaged the percent cover estimates of shrubs, trees, diversity indices, and large woody debris for all the vegetation plots within a given territory. We divided shrub cover into two categories: nutrient-rich shrub species and other shrub species. Nutrient-rich shrub species at our sites were salmonberry and stink currant, which are associated with riparian borders, categorized as nitrophiles, and indicate nitrogen-rich soil [18].

To determine forest stand characteristics, we recorded each tree species and its diameter at breast height (DBH) within a 11.3 m radius (400 m^2^ area surveyed) of the centre of each vegetation plot [49]. Tree species that occurred in less than five percent of plots were excluded from analyses. DBH was used to calculate the stand basal area (SBA) of all tree species, including western redcedar, Sitka spruce, and western hemlock, which dominate the forest composition. We determined SBA of tree species at both the 11.3 m plot level and at the territory level. Territory-level tree volume of each species was calculated by summing the SBA of each individual species from all the vegetation plots within a territory and dividing by the total area of those surveyed plots (expressed as m^2^ hectare^-1^).

### Body condition

When color-banding wrens, we collected morphometric data to calculate scaled mass index (SMI) to characterize adult male wren body condition. SMI is a condition index for estimating individual energy reserves standardized to body size as it accounts for the scaling relationship between mass and length [50]. We used wing length for our linear measurement as together with body mass, these measurements have been shown to predict percent body fat and fat mass in birds [51]. We also confirmed that wing measurements correlated strongly with adult wren mass. We calculated SMI as the predicted body mass for each individual when wing length is standardized by the arithmetic mean of the wing length of our measured adult wren population (46.52 mm) to the power of the slope of the regression [51].

### Data analysis

To test the hypothesis that salmon biomass may influence wren territory size, we used parameters of interest, such as salmon biomass, high-nutrient shrub cover, and coniferous tree volume as predictor variables for territory size. Parameters were selected based on previous literature showing that salmon and conifer composition influenced wren densities [24] and from observations of wrens using high-nutrient shrubs. We did not include other stream characteristics, as previous research at the same streams found watershed size characteristics that included width and length of the streams, and catchment size, explained little of the variation in Pacific Wren densities [24]. We used linear mixed effect-models with stream site as a random effect and year as a two-factor fixed effect in all candidate models. This resulted in a candidate set of seven models, including a null model with just year and stream (See S2 Table for full candidate model set).

We modeled male proportional countersinging rate (relative to singing rate) as a linear mixed-effect model with stream site as a random effect and year as a two-factor fixed effect. We included male stream-level absolute density, salmon biomass, and territory size as predictors of competition, resulting in a candidate model set of four.

To characterize habitat selection by wrens for our third study objective, we first overlayed the vegetation plots and 95% MCPs representing wren territories in order to determine if wrens are more likely to select habitat with specific vegetation characteristics. To model habitat selection, we created a resource selection probability function (RSPF) and competed generalized linear mixed-effect models with a binomial response of wren territory presence/absence (if the vegetation plot fell within or outside the 95% MCP) and logit link. We modeled the probability of wren territorial habitat selection as a function of vegetation characteristics, total salmon biomass in the stream, and year as predictors with stream as a random effect (see S3 Table for full candidate model set). We selected habitat parameters based on our prediction that wrens would prefer nitrophilic species such as salmonberry [18], complex habitat with lot of cover [42], and avoid forest stands with high volumes of western redcedar based on our observations. Year (2015 and 2016) was included as a two-level factor in all candidate models to account for year-to-year variability, however removing year from the models did not alter any predictions. Stream was included as a random effect in all models to help account for the potential confounding factors of other stream-level characteristics. We then competed 13 RSPF candidate models to determine a top model from which to generate predictions of habitat selection. The candidate models can be classified under four main hypotheses: 1) habitat only models, 2) salmon only models 3) salmon and habitat characteristics models and 4) salmon and habitat interaction models (S3 Table). This allowed us to test the hypothesis that salmon biomass and vegetation characteristics would interact to determine habitat selection.

Global models were assessed for violated assumptions using simulated residuals in the Dharma package [52]. For our RSPF models, absence of spatial autocorrelation was checked graphically using variograms. Vegetation plots were categorized as being within a wren territory if any part of them fell within the 95% minimum convex polygon of a territory.

As we had a limited sample size of banded wrens that we measured and weighed (half of the territorial males *n* = 27), we were only able to run simplified models to test for correlates of wren body condition. We modeled body condition as a function of territory size and territory size relative to others in the same stream, to test whether males with higher body condition had territories that were larger in absolute terms or larger than average within streams. We then competed these models with null models of only stream and year.

All candidate model predictors (including RSPF models and linear mixed-effect models) were checked for multicolinearity using variance inflation factor scores (VIF). None of the predictor variables had VIF scores greater than 2, indicating an acceptable amount of covariance [53] (see S3 Fig for correlation coefficients). Additionally, we standardized all explanatory variables (mean = 0, SD = 2) to allow for direct comparisons of fixed effects [54,55]. Using an information theoretic approach, we evaluated the relative support for each candidate model set and selected our top models using Akaike’s Information Criterion (AIC) with a cut-off of delta 2 AIC [56]. Territory size and countersinging top models were evaluated using delta AICc corrected for small sample size. All of our top models were > 2 AIC or > 2 AICc higher than the next highest ranked models, and were therefore not model averaged [56]. Wren density was modeled as a simple linear regression of salmon biomass. All statistical analyses were performed in R 3.4.1 [57].

## Results

We collected territory size and countersigning data from 44 male wrens from 11 streams in 2015 and 2016. This included data for 22 males in 2015, 11 of which were banded, and 22 different males in 2016, of which we successfully banded and collected measurements for 16. Thus, we banded a total of 27 male wrens. We also collected vegetation data at each stream from a total of 616 vegetation plots on which we based our habitat selection models.

**Fig 2:**
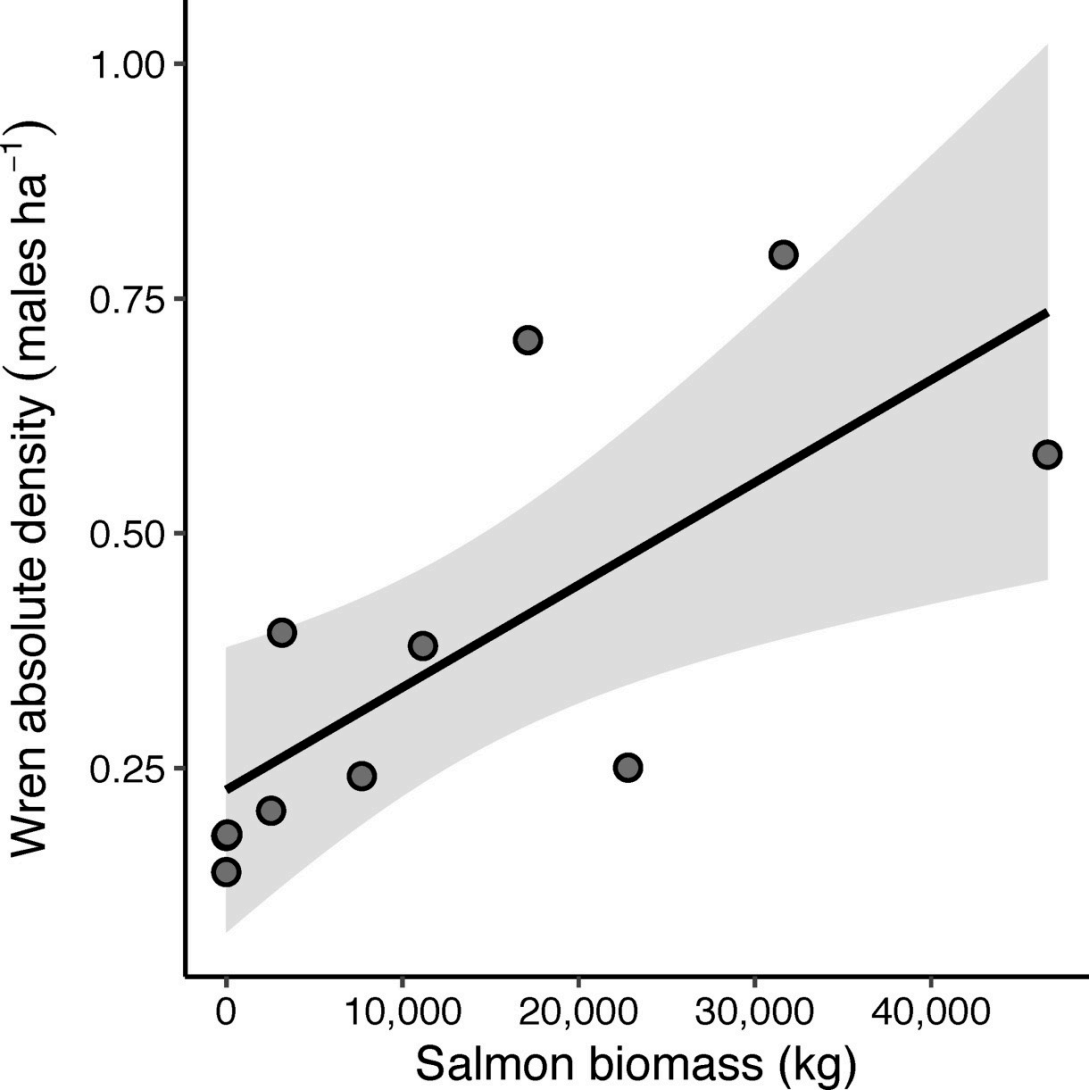
Relationship between salmon biomass per stream and absolute male Pacific wren density in the surrounding forest plot. The dark line represents the best-fit line from the linear model and the band represents the 95% confidence interval.

We confirmed our expectation that wren densities would be higher along streams with high salmon biomass (Fig 2). Wren absolute densities ranged from 0.1 to 0.8 wrens per hectare, with the highest salmon biomass stream having over 4 times higher average wren density than streams without salmon (P = 0.01, r^2^ = 0.53).

**Fig 3:**
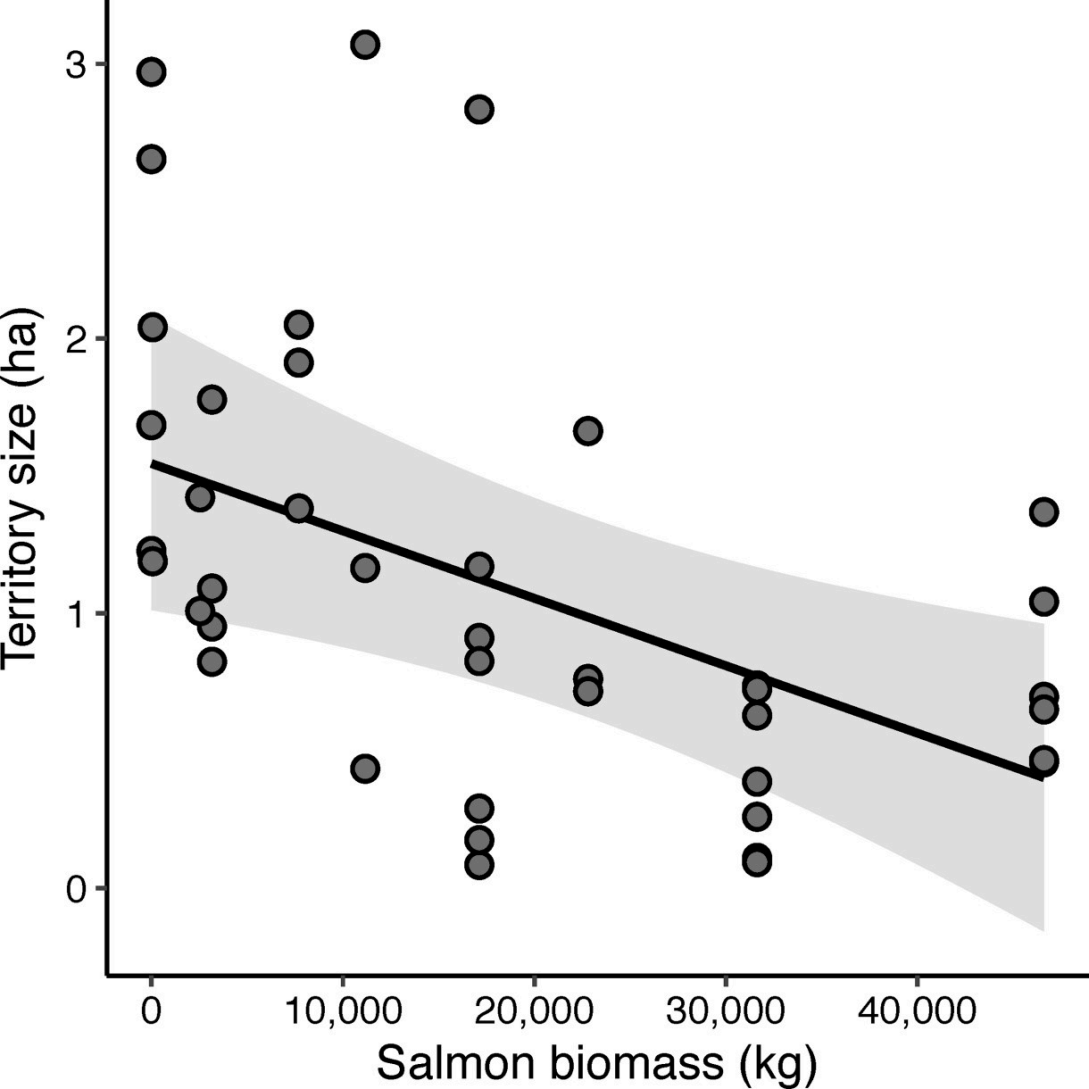
Relationship between the spawning salmon biomass at each stream and male wren territory size in surrounding habitat. The solid line represents the best fit from the top linear mixed-effect model for territory size with all other variables held at their mean. Each point represents an individual male wren’s territory size. The band represents the 95% confidence interval.

As we predicted, there was a negative relationship between wren territory size and salmon biomass (Fig 3). A two standard deviation increase in salmon biomass (31,663 kg) corresponded with a 67% (0.77 ± 0.55 hectare) reduction in male territory size. Salmon biomass was the most important predictor of territory size (Table 1). Surprisingly, none of our habitat measurements (e.g. high-nutrient shrub cover), were in the top model for territory size and habitat variables only accounted for a small amount of additional variation compared with salmon biomass alone.

**Table 1:** AICc model selection analysis of linear regressions (models presented: ΔAICc <5) describing Pacific wren territory size, countersinging rate, and body condition response variables predicted by salmon biomass, wren density and environmental metrics. Year (fixed effect) and stream (random effect) were included as variables in all models but are excluded from the table for clarity.

response	parameters	K	ΔAICc	w_i_	R^2^
territory size	salmon	3	0	0.51	0.37
	salmon + conifer cover	4	2.2	0.17	0.45
	null	2	3.0	0.12	0.41
	Salmon + riparian shrub cover	4	3.4	0.09	0.36
	Salmon + riparian shrub cover + conifer cover	5	4.7	0.05	0.45
proportional countersinging rate	male density	3	0	0.63	0.71
	salmon	3	2.4	0.19	0.71
	male density + salmon	4	3.7	0.10	0.71
	null	2	3.9	0.09	0.73
body condition	null	2	0	0.48	0.39
	relative territory size	3	0.4	0.41	0.46
	territory size	3	2.9	0.11	0.47

Body condition appears twice as due to limited sample size, we were only able to run simplified models to test for correlates of wren body condition and we modeled body condition as a function of territory size and relative territory size separately. K = number of parameters in model, ΔAICc = difference between the model AICc and the top model AICc, w_i_ = model AICc weight, R^2^ = regression coefficient of fitted versus observed values for each analysis, salmon = summed chum and pink salmon biomass, conifer cover = percent cover of all coniferous trees, male density = absolute male wren density per hectare, territory size = male wren territory size in hectares, null = model with only year (fixed effect) and stream (random effect).

**Fig 4:**
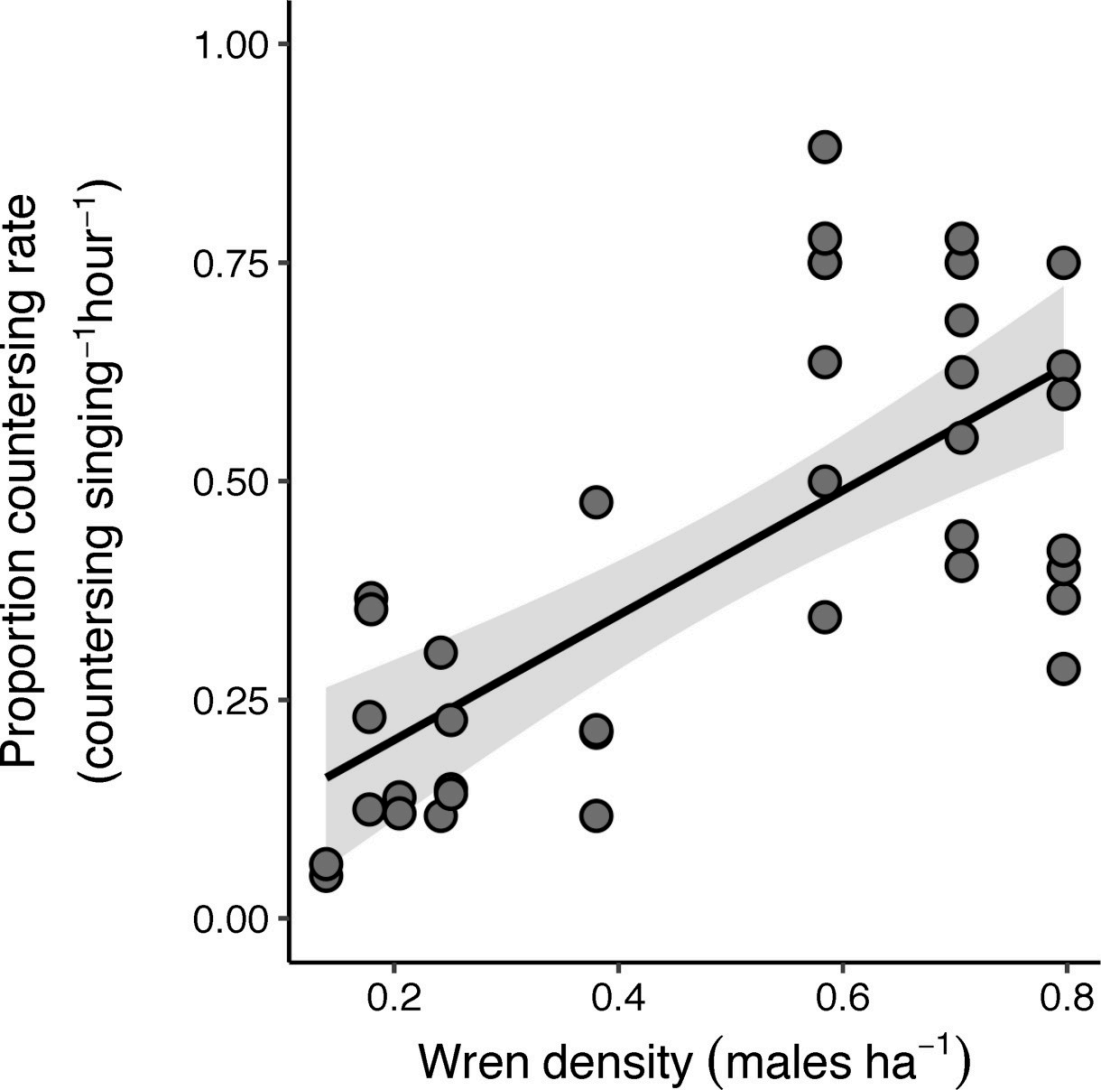
Relationship between absolute male wren density at each stream site and the individual male wren proportional rate of countersinging (countersinging divided by total singing bouts per hour). Wren density was the main predictor variable for the model. The solid line represents the best-fit line from the top model linear regression for proportional countersinging rate and the band represents the 95% confidence interval.

Individual males at streams with higher absolute wren densities had a higher proportional countersinging rate relative to singing rate than males at streams with low wren densities (Fig 4). On average, wrens at the highest density streams countersang at a 5.6 times higher rate than those at the lowest density wren streams (Fig 4). Wren density was the only important predictor of proportional countersinging rate (Table 1), and as territory size and wren density are highly correlated (r^2^ = 0.53), we chose wren density as a predictor variable to model proportional countersinging rate as it more closely reflects *in situ* competition.

**Fig 5:**
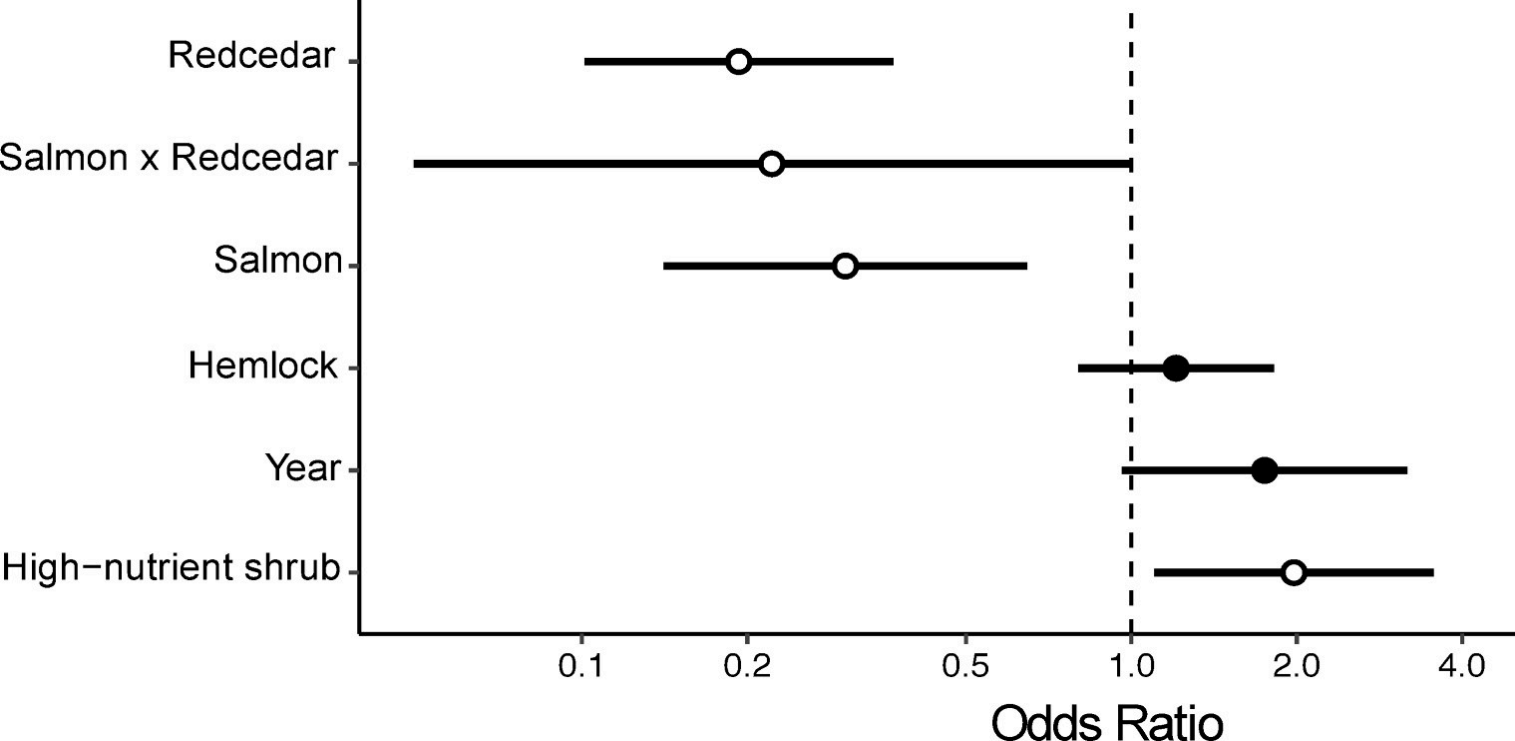
Odds ratio of habitat selection by territorial male wrens compared to habitat vegetation characteristics and stream-level spawning salmon biomass from the top AIC model. The standardized (mean = 0, SD = 2) parameters include redcedar stand basal area (m^2^ ha^-1^), salmon biomass (kg), their interaction, hemlock stand basal area (m^2^ ha^-1^), year, and percent high-nutrient shrub cover (stink currant and salmonberry). Circles show odds ratios for each parameter, with 95% confidence intervals indicated by horizontal lines. The odds ratios significantly different from 1 (95% CI do not overlap 1) are indicated by open circles.

Although habitat characteristics did not predict territory size, they did predict the locations that the birds defended along the streams. Hemlock tree volume, high-nutrient shrub cover, stream-level salmon biomass, and redcedar volume along with their interaction predicted wren habitat selection and were in the top model (Table 2). However, only high-nutrient shrub cover, redcedar volume, and salmon biomass, along with the interaction between salmon biomass and redcedar volume had a statistically significant relationship with probability of occupation with parameter estimates not overlapping zero (Fig 5). Diversity of shrubs and trees were not important predictors of habitat selection.

**Table 2:** AIC model selection from the logistic regression models for probability of habitat selection by territorial male wrens (models presented: ΔAIC <5). Year (fixed effect) and stream (random effect) were included as variables in all models but are excluded from the table for clarity. The full candidate model set is described in S1 Table.

response	parameters	K	ΔAIC	w_i_	ER
probability of habitat selection	salmon * redcedar + high-nutrient shrub + hemlock	7	0	0.61	1.00
	salmon * high-nutrient shrub + other shrub + hemlock	8	2.0	0.23	2.68
	salmon + redcedar + high-nutrient shrub + other shrub + hemlock	7	3.8	0.09	6.75

Habitat parameters tested include: Salmon = summed chum and pink salmon biomass per stream (kg), redcedar = western redcedar stand basal area (m^2^ ha^-1^), high-nutrient shrub = percent salmonberry and stink currant shrub cover, hemlock = western hemlock stand basal area (m^2^ ha^-1^), and other shrub = percent cover of blueberry, false azalea, and salal. K = number of parameters in model, ΔAIC = difference between the model AIC and the top model AIC, w_i_ = model AIC weights, ER = evidence ratio (relative likelihood of top model compared to given model).

**Fig 6:**
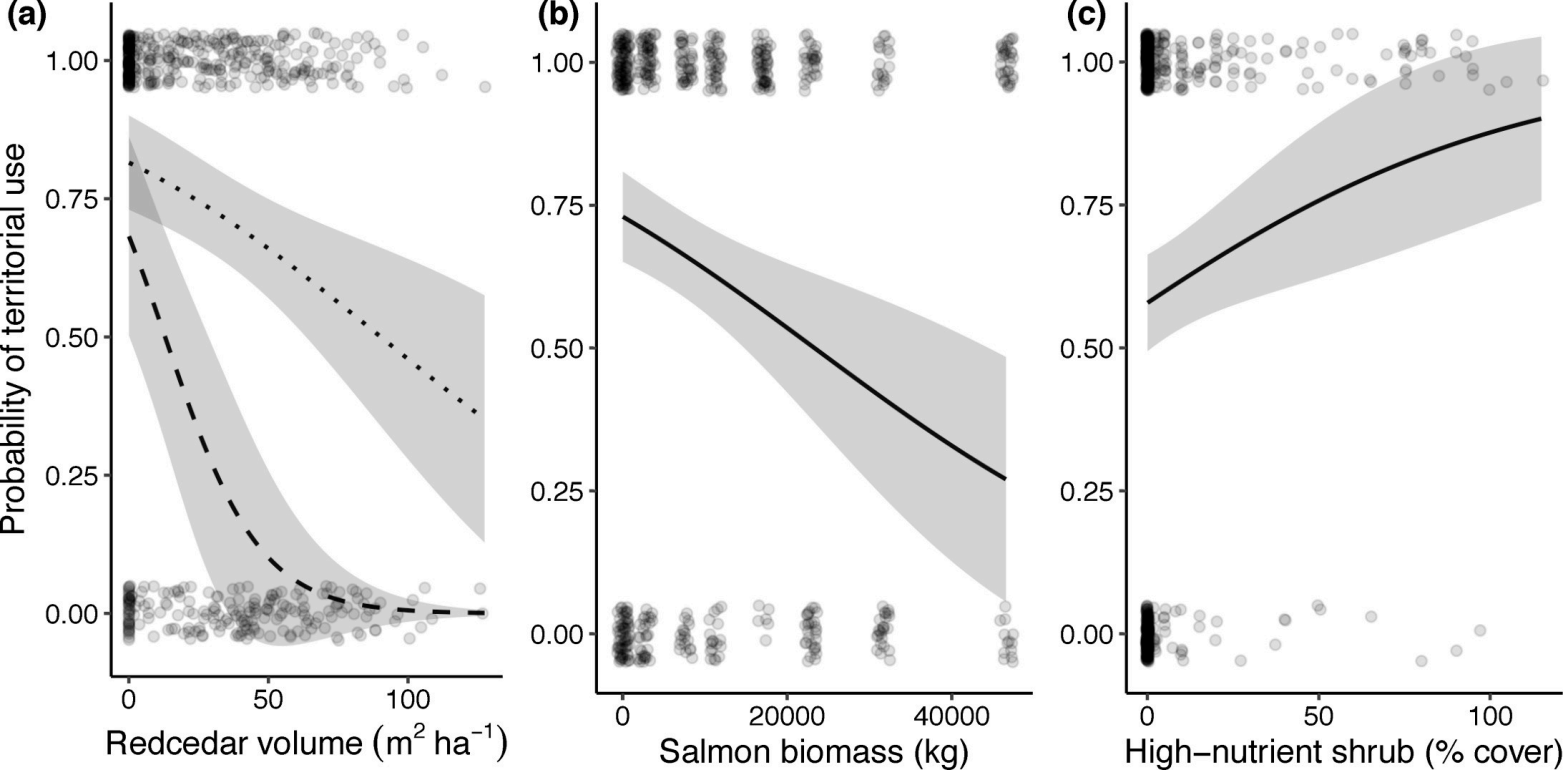
Resource selection probability plots for important predictor variables for the top model of probability of territorial use by male wrens. Lines are predicted probabilities of habitat being included in wren territories against (a) western redcedar stand basal area (m^2^ ha^-1^) at high salmon biomass (47,000 kg, represented by the dashed line) and no salmon (0 kg, represented by the dotted line), (b) spawning salmon biomass (kg) at stream-level, and (c) percent high-nutrient shrub cover, with other variables set to their mean value. Bands are 95% confidence intervals and points are jittered to display the spread of data.

The resource selection probability function plots further supported our findings that wrens preferred locations with more high-nutrient shrub cover and lower volumes of western redcedar, however wrens were more likely to avoid redcedar at high salmon biomass streams (Fig 6). Wrens were 46% less likely to select habitat with high volumes of western redcedar at streams with no salmon, however that rose to 68% lower likelihood at streams with high salmon biomass. Additionally, wrens were 32% more likely to select habitat with more high-nutrient shrub cover across all streams.

Wrens were also less likely to select habitat along streams that had more salmon, with the odds of wrens selecting available habitat at a higher salmon biomass stream were less than one third that at a low salmon biomass stream (Fig 5). We tested this explicitly by analysing the difference between defended and undefended surveyed habitat and found evidence to suggest that as total salmon biomass increases, the amount of undefended habitat also increases in riparian forests (P = 0.056, r^2^ = 0.35; see S4 Fig).

We found that neither territory size, nor having a larger territory size relative to others along the same stream, weregood predictors of adult male wren body condition (Table 1). Wrens with larger territories both across streams and relative to their neighbours had similar scaled mass indices. The inclusion of territory size or relative territory size as predictor variables did not improve model fit relative to the null model (Table 1).

## Discussion

This study presents evidence to suggest that nutrients from salmon carcasses shape trade-offs of territoriality for a species that does not directly feed on salmon, leading to higher densities along salmon streams. Specifically, streams with higher salmon biomass during the fall spawning season support higher absolute male Pacific wren densities in the spring and summer (Fig 2), corresponding with a decrease in wren territory size (Fig 3). At streams with higher densities of birds and higher salmon biomass there was increased competition between male wrens as shown by higher rates of countersinging (Fig 4). Wrens were more selective of what habitat they defended on salmon streams; they were more likely to avoid western redcedar on high salmon biomass streams (Fig 6) perhaps because the costs of defending territories may be higher on streams with high wren densities. While there may be a higher cost of defending lower quality habitat at streams with high densities of wrens, our data show no clear relationship between territory size and changes in adult male body condition (Table 1).

Salmon nutrients may be shaping territory size through several indirect pathways [58]. Salmon streams may provide increased food availability for wrens, as previous studies have shown that nutrient subsidies from salmon carcasses produce higher insect biomass and affect riparian plant community composition [18,38,59–61]. Higher food availability has been shown to have an inverse relationship with territory size across many species [62–64], which may allow higher densities of wrens on salmon streams [23]. Additionally, higher complexity of habitat structure through foliage density or high shrub density has been shown to correlate with decreased territory sizes of bird species [35,65]. As many animals use food and habitat structure as proximate cues of habitat quality, these measures allow animals to scale their territory sizes with available resources [35,45,66].

Although we hypothesized that salmon biomass would be correlated with wren territory size, we were surprised that it was the only predictor from the many vegetation characteristics that we measured. Thus, contrary to other studies, no other habitat variables that we measured, including high-nutrient shrub cover, conifer cover or plant species richness were an important influence on wren territory size [26,42,67]. Vegetation characteristics alone may not be the cues that wrens use to determine territory size. Salmon biomass may influence other measures of habitat quality, including increased food availability [17,38]. Salmon subsidies may also improve both the quantity and quality of available resources by increasing shrub quality for herbivorous insects, such as caterpillars, which are an important component of wren diet [68–70].

The countersinging data indicate that along streams with high wren densities, individual male wrens compete directly with conspecifics more than those in low density habitats (Fig 4). Competition represents a direct energy cost, as males are able to allocate less time to foraging and provisioning young [48,71,72]. Competition can reduce territory size, which in turn can lead to a reduction in individual fitness [73]. When competition is reduced experimentally by reducing competitor density, male great tits (*Parus major*) responded by increasing territory size, resulting in higher numbers of second generation recruits [73]. Indeed, some studies have shown that cost of defense is more important in determining territory characteristics than resource availability [37,74]. We found that despite having higher density of wrens with smaller territories at high salmon biomass streams, there is also more undefended habitat. This suggests that competition keeps wrens from being uniformly distributed across the available habitat, as there may be a higher cost associated with defending low-quality habitat (Figs 6 and S4). Thus, we suggest that the high rate of countersinging necessary to defend territories at high-density streams may structure trade-offs that affect territory selection at high salmon biomass streams.

There may be a higher cost of defending low-quality habitats at high wren density streams due to greater competition with conspecifics. We found that wrens select habitat based on specific vegetation characteristics across streams, specifically avoiding western redcedar and selecting for high-nutrient shrub cover. However, wrens are more likely to avoid habitat with redcedar on streams with more salmon nutrient input, where wrens achieve higher densities (Fig 6). Redcedar may represent lower quality vegetation as there are fewer insects on redcedar compared with deciduous shrubs or other trees due to monoterpenes contained in their foliage which deter oviposition and insect herbivory [20,75]. Wrens typically forage low in the understory and glean insects from bark and twigs, so redcedar would not be ideal foraging habitat [76]. Salmonberry and stink currant, conversely, are often used for more camouflaged nest site locations [26] and they are associated with high nitrogen availability [18] which in turn may improve both quality and quantity of insect prey [69,77].

Wrens at high densities along high salmon biomass streams do not have higher body condition than those at low salmon biomass streams. Previous studies have found that higher resource availability can increase individual body condition [78,79]. However, the benefits associated with higher quality habitat may be balanced by the negative effects of high competition. This trade-off at high salmon streams may result in wrens experiencing a lower realized habitat quality, or the habitat quality the competitor actually experiences [32,34]. Wrens having equal body condition across heterogeneous habitats suggests that they optimize their territory size to balance different pressures in ecosystems with differing amounts of resources and competition.

In conclusion, this study suggests that marine-derived nutrients from salmon carcasses indirectly affect summer territorial behaviour by an avian insectivore. Further research would be useful to inform dispersal patterns of fledglings and whether salmon-derived nutrients are correlated with overwinter survival and dispersal of juveniles, which is an important population limitation in songbirds [80]. However, in the breeding season, along streams with high salmon biomass, we observed higher densities of Pacific wrens and males defending smaller territories. In turn, there seems to be increased competition along streams with more salmon, as evidenced by higher rates of countersinging. Increased competition may be responsible for the birds avoiding defence of lower quality habitat [32,34] Thus, this correlational study suggests that the nutrient subsidy provided by salmon appears to shift the economics of territorial defence towards smaller, highly-contested territories in high-quality habitat, leading to higher densities of wrens.

## Supporting information

S1 Fig The relationships between salmon biomass (in kg) per stream and stream width (in meters). (PDF)

S2 Fig Schematic of how vegetation plots were set up at each stream site using a modified BBIRD protocol developed by Martin (1997) [49]. Small green circles represent the 5 m radius plots where we measured percent cover of trees under five meters height, shrubs cover, logs and stump over and calculated Shannon’s Diversity of tree and shrub species. Percent cover was estimated by trained observers using datasheets to sketch out percent cover of each species on circle divided into quadrants (see detail inset). The dotted line circles represent the 11.3 m radius plots (400 m^2^ area surveyed) that we used to determine forest stand characteristics. We recorded the DBH of all tree species within this plot and calculated the stand basal area of all tree species.
(PNG)

S3 Fig The correlation coefficients for all variables used in candidate model sets shown with numbers and represented using colour. Circle size represents strength of correlation and circle colour represents direction (blue = positive, red = negative). Parameters include: Alder = red alder stand basal area (m^2^ ha^-1^), conifer cover = percent cover of small conifer trees, density = male wren density (males ha^-1^), hemlock = western hemlock stand basal area (m^2^ ha^-1^), high-nutrient shrubs = percent salmonberry and stink currant shrub cover, logs and stumps = percent cover of all large woody debris, other shrub = percent cover of shrubs blueberry, false azalea, and salal, redcedar = western redcedar stand basal area (m^2^ ha^-1^), salmon = summed chum and pink salmon biomass per stream (kg), shrub diversity = Shannon diversity index of all shrub species, spruce = Sitka spruce stand basal area (m^2^ ha^-1^), tree diversity = Shannon diversity index of all tree species.
(PDF)

S4 Fig The relationships between salmon biomass per stream and undefended habitat not included within wren territories in the surrounding forest plot. The dark line represents the best-fit line and the band represents the 95% confidence interval.
(PDF)

S1 Table Stream characteristics and salmon population data (2012–2015) for watersheds (n = 11) in this study. (PDF)

S2 Table AICc model selection analysis of linear regressions (full candidate model sets) describing Pacific wren territory size, countersinging rate, and body condition response variables predicted by salmon biomass, wren density and environmental metrics. Habitat parameters tested include: Salmon = summed chum and pink salmon biomass, conifer cover = percent cover of all coniferous trees, riparian shrub cover = percent cover of riparian shrubs predominantly stink currant and salmonberry, male density = absolute male wren density per hectare, territory size = male wren territory size in hectares. Year (fixed effect) and stream (random effect) were included as variables in all models but are excluded from the table for clarity. K = number of parameters in model, ΔAICc = difference between the model AICc and the top model AICc, w_i_ = model AICc weight, ER = evidence ratio.
(PDF)

S3 Table All candidate models from the logistic regression models for probability of habitat selection by territorial male wrens. Habitat parameters tested include: Salmon = summed chum and pink salmon biomass per stream (kg), high-nutrient shrubs = percent salmonberry and stink currant shrub cover, other shrubs = percent cover of shrubs blueberry, false azalea, and salal, conifer cover = percent cover of small conifer trees, shrub diversity = Shannon diversity index of all shrub species, hemlock = western hemlock stand basal area (m^2^ ha^-1^), redcedar = western redcedar stand basal area (m^2^ ha^-1^), alder = red alder stand basal area (m^2^ ha^-1^), spruce = Sitka spruce stand basal area (m^2^ ha^-1^), tree diversity = Shannon diversity index of all tree species, logs and stumps = percent cover of all large woody debris. Year (fixed effect) and stream (random effect) were included as variables in all models but are excluded in the table for clarity. K = number of parameters in model, ΔAIC = difference between the model AIC and the top model AIC, w_i_ = model AIC weights, ER = evidence ratio.
(PDF)

## References

[1] Polis GA, Anderson WB, Holt RD. Towards an integration of landscape and food web ecology: the dynamics of spatially subsidized food webs. Annu Rev Ecol Syst. 1997;28(1):289–316.

[2] Bentley KT, Schindler DE, Armstrong JB, Zhang R, Ruff CP, Lisi PJ. Foraging and growth responses of stream-dwelling fishes to inter-annual variation in a pulsed resource subsidy. Ecosphere. 2012;3(12):1–17.

[3] Anderson WB, Wait DA, Stapp P. Resources from another place and time: responses to pulses in a spatially subsidized system. Ecology. 2008;89:660–670. doi: 10.1890/07-0234.1 18459330

[4] Weber MJ, Brown ML. Continuous, pulsed and disrupted nutrient subsidy effects on ecosystem productivity, stability, and energy flow. Ecosphere. 2013;4(2):1–13.

[5] Holt RD, Barfield M. Impacts of temporal variation on apparent competition and coexistence in open ecosystems. Oikos. 2003;101(1):49–58.

[6] Bartels P, Cucherousset J, Steger K, Eklöv P, Tranvik LJ, Hillebrand H. Reciprocal subsidies between freshwater and terrestrial ecosystems structure consumer resource dynamics. Ecology. 2012;93(5):1173–82. doi: 10.1890/11-1210.1 22764503

[7] Huxel GR, McCann K, Polis GA. Effects of partitioning allochthonous and autochthonous resources on food web stability. Ecol Res. 2002;17(4):419–32.

[8] Nakano S, Murakami M. Reciprocal subsidies: dynamic interdependence between terrestrial and aquatic food webs. Proc Natl Acad Sci U S A. 2001;98(1):166–70. doi: 10.1073/pnas.98.1.166 11136253 PMC14562

[9] Jefferies RL. Allochthonous inputs: integrating population changes and food-web dynamics. Trends Ecol Evol. 2000;15(1):19–22. doi: 10.1016/s0169-5347(99)01758-9 10603500

[10] Gratton C, Denno RF. Inter-year carryover effects of a nutrient pulse on Spartina plants, herbivores, and natural enemies. Ecology. 2003;84(10):2692–707.

[11] Ostfeld RS, Keesing F. Pulsed resources and community dynamics of consumers in terrestrial ecosystems. Trends Ecol Evol. 2000;15(6):232–7. doi: 10.1016/s0169-5347(00)01862-0 10802548

[12] Richardson JS, Sato T. Resource subsidy flows across freshwater-terrestrial boundaries and influence on processes linking adjacent ecosystems. Ecohydrology. 2015;8(3):406–15.

[13] Quinn TP, Carlson SM, Gende SM, Rich HB Jr. Transportation of Pacific salmon carcasses from streams to riparian forests by bears. Can J Zool. 2009;87(3):195–203.

[14] Darimont CT, Reimchen TE, Paquet PC. Foraging behaviour by gray wolves on salmon streams in coastal British Columbia. Can J Zool. 2003;81(2):349–53.

[15] Buxton TH, Buffington JM, Tonina D, Fremier AK, Yager EM. Modeling the influence of salmon spawning on hyporheic exchange of marine-derived nutrients in gravel stream beds. Can J Fish Aquat Sci. 2015;72(8):1146–58.

[16] Andersson LC, Reynolds JD. Effects of habitat features on size-biased predation on salmon by bears. Oecologia. 2017;184(1):101–14. doi: 10.1007/s00442-017-3845-0 28251344

[17] Gende SM, Edwards RT, Willson MF, Wipfli MS. Pacific salmon in aquatic and terrestrial ecosystems. Bioscience. 2002;52(10):917.

[18] Hocking MD, Reynolds JD. Impacts of salmon on riparian plant diversity. Science. 2011;331(6024):1609–12. doi: 10.1126/science.1201079 21442794

[19] Helfield JM, Naiman RJ. Effects of salmon-derived nitrogen on riparian forest growth and implications for stream productivity. Ecology. 2001;82(9):2403–9.

[20] Allan JD, Wipfli MS, Caouette JP, Prussian A, Rodgers J. Influence of streamside vegetation on inputs of terrestrial invertebrates to salmonid food webs. Can J Fish Aquat Sci. 2003;60(3):309–20.

[21] Gende SM, Willson MF. Passerine densities in riparian forests of southeast Alaska: potential effects of anadromous spawning salmon. Condor. 2001;103(3):624–9.

[22] Field RD, Reynolds JD. Sea to sky: impacts of residual salmon-derived nutrients on estuarine breeding bird communities. Proc Biol Sci. 2011;278(1721):3081–8. doi: 10.1098/rspb.2010.2731 21325324 PMC3158931

[23] Christie KS, Reimchen TE. Presence of salmon increases passerine density on pacific northwest streams. Auk. 2008;125(1):51–9.

[24] Wagner MA, Reynolds JD. Salmon increase forest bird abundance and diversity. PLoS One. 2019;14(2):e0210031. doi: 10.1371/journal.pone.0210031 30726212 PMC6364887

[25] Uesugi A, Murakami M. Do seasonally fluctuating aquatic subsidies influence the distribution pattern of birds between riparian and upland forests? Ecol Res. 2007;22(2):274–81.

[26] De Santo TL, Willson MF, Bartecchi KM, Weinstein J. Variation in nest sites, nesting success, territory size, and frequency of polygyny in winer wrens in northern temperate coniferous forests. Wilson Bull. 2003;115:29–37.

[27] Pearson SF, Manuwal DA. Breeding bird response to riparian buffer width in managed pacific northwest Douglas-fir forests. Ecol Appl. 2001;11(3):840–53.

[28] Christie KS, Hocking MD, Reimchen TE. Tracing salmon nutrients in riparian food webs: isotopic evidence in a ground-foraging passerine. Can J Zool. 2008;86(11):1317–23.

[29] Marczak LB, Thompson RM, Richardson JS. Meta-analysis: trophic level, habitat, and productivity shape the food web effects of resource subsidies. Ecology. 2007;88(1):140–8. doi: 10.1890/0012-9658(2007)88[140:mtlhap]2.0.co;2 17489462

[30] McLoughlin PD, Lysak K, Debeffe L, Perry T, Hobson KA. Density-dependent resource selection by a terrestrial herbivore in response to sea-to-land nutrient transfer by seals. Ecology. 2016;97(8):1929–37. doi: 10.1002/ecy.1451 27859192

[31] Van Horne B. Density as a misleading indicator of habitat quality. J Wildl Manage. 1983;47(4):893–901.

[32] Johnson MD. Measuring habitat quality: a review. Condor. 2007;109(3):489–507.

[33] Jones J. Habitat selection studies in avian ecology: a critical review. Auk. 2001;118(2):557–62.

[34] Jones JA, Harris MR, Siefferman L. Physical habitat quality and interspecific competition interact to influence territory settlement and reproductive success in a cavity nesting bird. Front Ecol Evol. 2014;2:1–8.

[35] Marshall MR, Cooper RJ. Territory size of a migratory songbird in response to caterpillar density and foliage structure. Ecology. 2004;85(2):432–45.

[36] Stenger J. Food habits and available food of ovenbirds in relation to territory size. Auk. 1958;75:335–346.

[37] Adams ES. Approaches to the study of territory size and shape. Annu Rev Ecol Syst. 2001;32(1):277–303.

[38] Hocking MD, Ring RA, Reimchen TE. The ecology of terrestrial invertebrates on Pacific salmon carcasses. Ecol Res. 2009;24(5):1091–100.

[39] Chesser RT, Banks RC, Barker FK, Cicero C, Dunn JL, Kratter AW, et al. Fifty-first supplement to the american ornithologists’ union check-list of North American birds. Auk. 2010;127:726–744.

[40] Pojar J, Klinka K, Meidinger DV. Biogeoclimatic ecosystem classification in British Columbia. For Ecol Manage. 1987;22(1–2):119–54.

[41] Bibby CJ, Burgess ND, Hill DA, Mustoe S. Bird Census Techniques. 2nd ed. San Diego, CA: Academic Press; 2000.

[42] Waterhouse L. Habitat of Winter Wrens in riparian and upland areas of coastal forests. [Burnaby, BC]: Simon Fraser University; 1998.

[43] English KK, Bocking RC, Irvine JR. A robust procedure for estimating salmon escapement based on the area-under-the-curve method. Can J Fish Aquat Sci. 1992;49(10):1982–9.

[44] Ralph CJ, Geupel J, Geoffrey R, Pyle P, Martin TE, DeSante DF. Handbook of field methods for monitoring landbirds. Albany, CA: Pacific Southwest Research Station, Forest Service, USDA; 1993.

[45] Odum EP, Kuenzler EJ. Measurement of territory and home range size in birds. Auk. 1955;72(2):128–37.

[46] Hyman J. Countersinging as a signal of aggression in a territorial songbird. Anim Behav. 2003;65(6):1179–85.

[47] Calenge C. The package adehabitat for the R software: a tool for the analysis of space and habitat use by animals. Ecol Modell. 2006;197:516–519.

[48] Sillett TS, Rodenhouse NL, Holmes RT. Experimentally reducing neighbor density affects reproduction and behavior of a migratory songbird. Ecology. 2004;85(9):2467–77.

[49] Martin TE, Paine C, Conway CJ, Hochachka WM, Allen P, Jenkins W. BBIRD field protocol. In Missoula, MT: Biological Resources Division, Montana Cooperative Wildlife Research Unit, University of Montana; 1997.

[50] Peig J, Green AJ. The paradigm of body condition: a critical reappraisal of current methods based on mass and length: The paradigm of body condition. Funct Ecol. 2010;24(6):1323–32.

[51] Peig J, Green AJ. New perspectives for estimating body condition from mass/length data: the scaled mass index as an alternative method. Oikos. 2009;118(12):1883–91.

[52] Hartig F. DHARMa: residual diagnostics package, hierarchical (multi-level/mixed) regression models. R. 2018.

[53] Zuur AF, Ieno EN, Walker N, Saveliev AA, Smith GM. Mixed effects models and extensions in ecology with R. New York, NY: Springer; 2009.

[54] Gelman A. Scaling regression inputs by dividing by two standard deviations. Stat Med. 2008;27(15):2865–73. doi: 10.1002/sim.3107 17960576

[55] Schielzeth H. Simple means to improve the interpretability of regression coefficients. Methods Ecol Evol. 2010;1(2):103–13.

[56] Burnham KP, Anderson DR. Information and likelihood theory: a basis for model selection and inference. In: Model Selection and Multimodel Inference. Springer New York; 2002. p. 49–97.

[57] R Core Team. R: A Language and Environment for Statistical Computing [Internet]. Vienna, Austria; 2016. Available from: https://www.R-project.org/.

[58] Reimchen TE. Diverse ecological pathways of salmon nutrients through an intact marine-terrestrial interface. Can F Nat. 2017;131:350–368.

[59] Hocking MD, Reynolds JD. Nitrogen uptake by plants subsidized by Pacific salmon carcasses: a hierarchical experiment. Can J For Res. 2012;42(5):908–17.

[60] Collins SF, Baxter CV. Heterogeneity of riparian habitats mediates responses of terrestrial arthropods to a subsidy of Pacific salmon carcasses. Ecosphere. 2014;5(11):1–14.

[61] Bilby RE, Fransen BR, Bisson PA. Incorporation of nitrogen and carbon from spawning coho salmon into the trophic system of small streams: evidence from stable isotopes. Can J Fish Aquat Sci. 1996;53(1):164–73.

[62] Kittle AM, Anderson M, Avgar T, Baker JA, Brown GS, Hagens J, et al. Wolves adapt territory size, not pack size to local habitat quality. J Anim Ecol. 2015;84(5):1177–86. doi: 10.1111/1365-2656.12366 25757794

[63] Dill LM, Ydenberg RC, Fraser AHG. Food abundance and territory size in juvenile coho salmon (Oncorhynchus kisutch). Can J Zool. 1981;59(9):1801–9.

[64] Hixon MA. Food production and competitor density as the determinants of feeding territory size. Am Nat. 1980;115(4):510–30.

[65] Conner RN, Anderson ME, Dickson JG. Relationships among territory size, habitat, song, and nesting success of Northern Cardinals. Auk. 1986;103(1):23–31.

[66] Tingley MW, Wilkerson RL, Howell CA, Siegel RB. An integrated occupancy and space‐use model to predict abundance of imperfectly detected, territorial vertebrates. Methods Ecol Evol. 2016;7(5):508–17.

[67] Evans Ogden LJ, Martin M, Martin K. Mating and breeding success decline with elevation for the pacific wren (Troglodytes pacificus) in coastal mountain forests. Wilson J Ornithol. 2012;124(2):270–6.

[68] Van Horne B, Bader A. Diet of nestling winter wrens in relationship to food availability. Condor. 1990;92(2):413.

[69] Marcarelli AM, Baxter CV, Mineau MM, Hall RO Jr. Quantity and quality: unifying food web and ecosystem perspecitves on the role of resource subsidies in freshwaters. Ecology. 2011;92:1215–1225. doi: 10.1890/10-2240.1 21797150

[70] Awmack CS, Leather SR. Host plant quality and fecundity in herbivorous insects. Annu Rev Entomol. 2002;47(1):817–44. doi: 10.1146/annurev.ento.47.091201.145300 11729092

[71] King DI, Chandler RB, Collins JM, Petersen WR, Lautzenheiser TE. Effects of width, edge and habitat on the abundance and nesting success of scrub–shrub birds in powerline corridors. Biol Conserv. 2009;142(11):2672–80.

[72] Qvarnström A. Experimentally increased badge size increases male competition and reduces male parental care in the collared flycatcher. Proc Biol Sci. 1997;264(1385):1225–31.

[73] Both C, Visser ME. Breeding territory size affects fitness: an experimental study on competition at the individual level: Population density, territory size and fitness. J Anim Ecol. 2008;69(6):1021–30.

[74] Eason P. Optimization of territory shape in heterogeneous habitats: A field study of the red-capped cardinal (Paroaria gularis). J Anim Ecol. 1992;61(2):411.

[75] Alfaro RI, Pierce HD Jr, Borden JH, Oehlschlager AC. Insect feeding and oviposition deterrents from western red cedar foliage. J Chem Ecol. 1981;7(1):39–48. doi: 10.1007/BF00988634 24420426

[76] Holmes RT, Robinson SK. Spatial patterns, foraging tactics, and diets of ground-foraging birds in a northern hardwoods forest. Wilson Bull. 1988;100:317–394.

[77] Hocking MD, Reimchen TE. Salmon-derived nitrogen in terrestrial invertebrates from coniferous forests of the Pacific Northwest. BMC Ecol. 2002;2:4. doi: 10.1186/1472-6785-2-4 11914157 PMC101382

[78] Kitaysky AS, Wingfield JC, Piatt JF. Dynamics of food availability, body condition and physiological stress response in breeding Black-legged Kittiwakes. Funct Ecol. 1999;13(5):577–84.

[79] Brown DR, Sherry TW. Food supply controls the body condition of a migrant bird wintering in the tropics. Oecologia. 2006;149(1):22–32. doi: 10.1007/s00442-006-0418-z 16639569

[80] Sillett TS, Holmes RT. Variation in survivorship of a migratory songbird throughout its annual cycle. J Anim Ecol. 2002;71(2):296–308.

