# Supplementary figures and images for: Salmon subsidies predict territory size and habitat selection of an avian insectivore
Source: PLoS One. 2021 Jul 8;16(7):e0254314. doi: 10.1371/journal.pone.0254314 (PMC8266124; doi:10.1371/journal.pone.0254314)

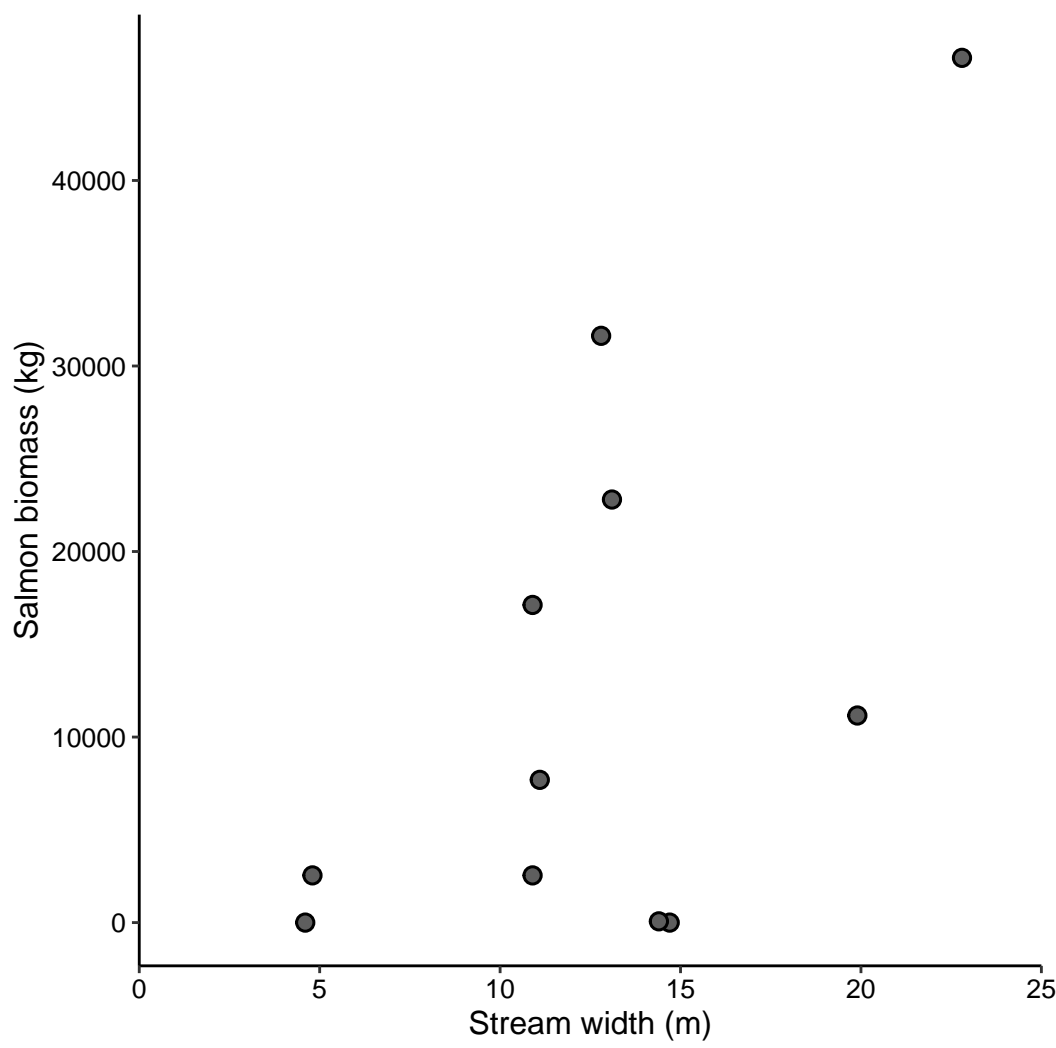

Supplement: S1 Fig — (PDF) [file pone.0254314.s001.pdf]

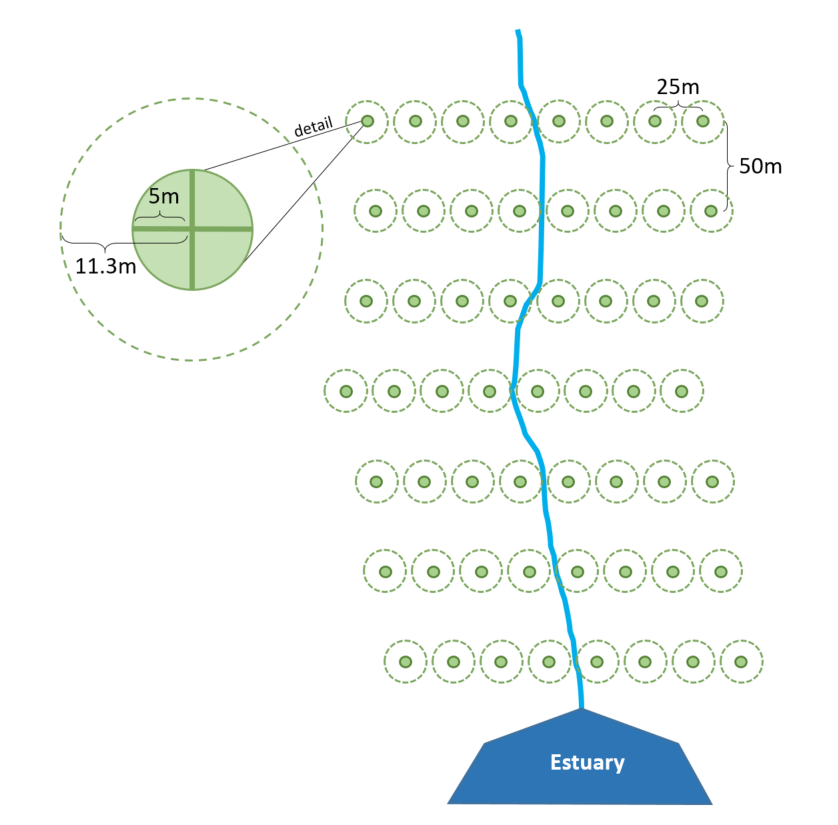

Supplement: S2 Fig — Small green circles represent the 5 m radius plots where we measured percent cover of trees under five meters height, shrubs cover, logs and stump over and calculated Shannon’s Diversity of tree and shrub species. Percent cover was estimated by trained observers using datasheets to sketch out percent cover of each species on circle divided into quadrants (see detail inset). The dotted line circles represent the 11.3 m radius plots (400 m2 area surveyed) that we used to determine forest stand characteristics. We recorded the DBH of all tree species within this plot and calculated the stand basal area of all tree species. (PNG) [file pone.0254314.s002.png]

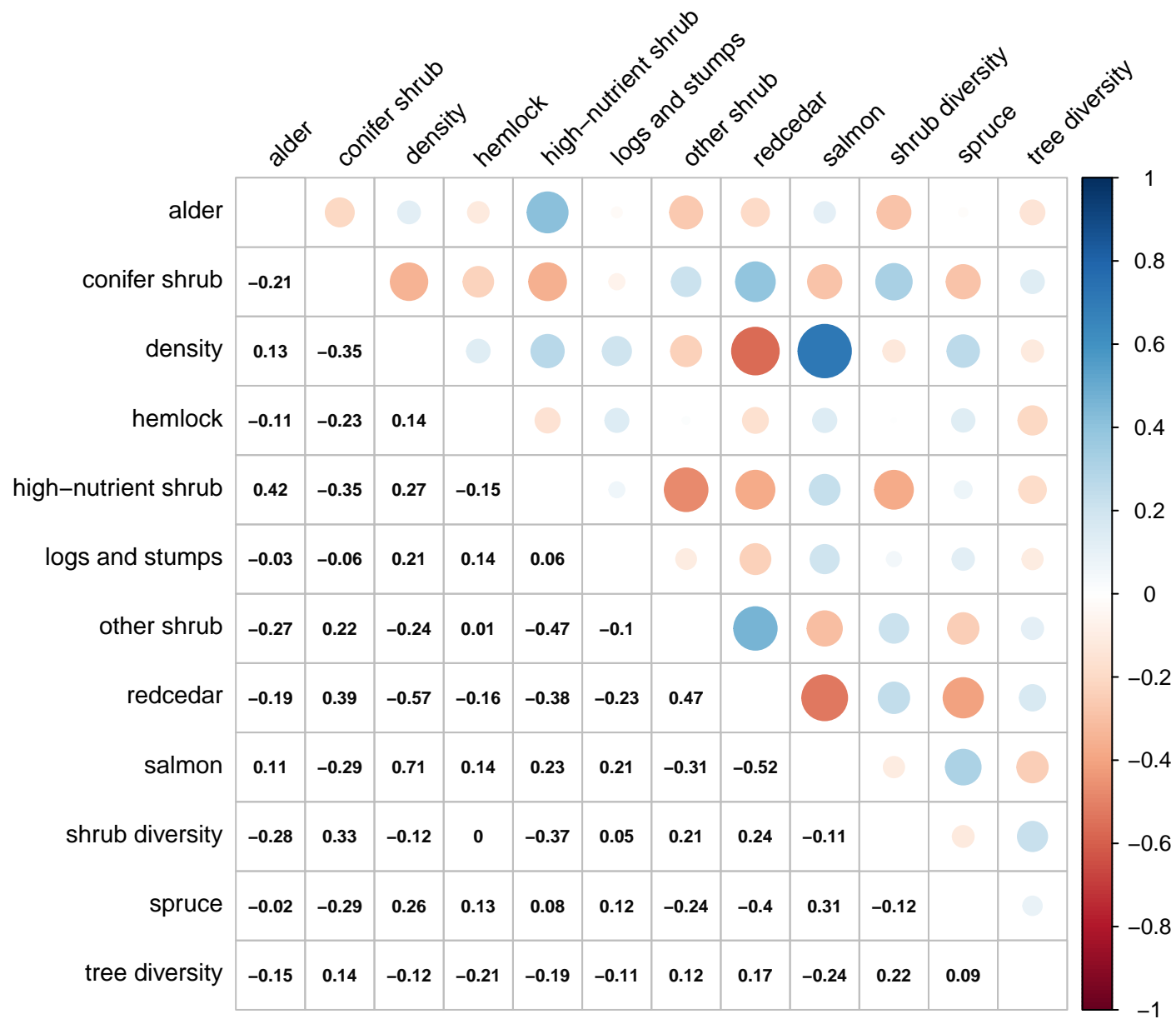

Supplement: S3 Fig — Circle size represents strength of correlation and circle colour represents direction (blue = positive, red = negative). Parameters include: Alder = red alder stand basal area (m2 ha-1), conifer cover = percent cover of small conifer trees, density = male wren density (males ha-1), hemlock = western hemlock stand basal area (m2 ha-1), high-nutrient shrubs = percent salmonberry and stink currant shrub cover, logs and stumps = percent cover of all large woody debris, other shrub = percent cover of shrubs blueberry, false azalea, and salal, redcedar = western redcedar stand basal area (m2 ha-1), salmon = summed chum and pink salmon biomass per stream (kg), shrub diversity = Shannon diversity index of all shrub species, spruce = Sitka spruce stand basal area (m2 ha-1), tree diversity = Shannon diversity index of all tree species. (PDF) [file pone.0254314.s003.pdf]

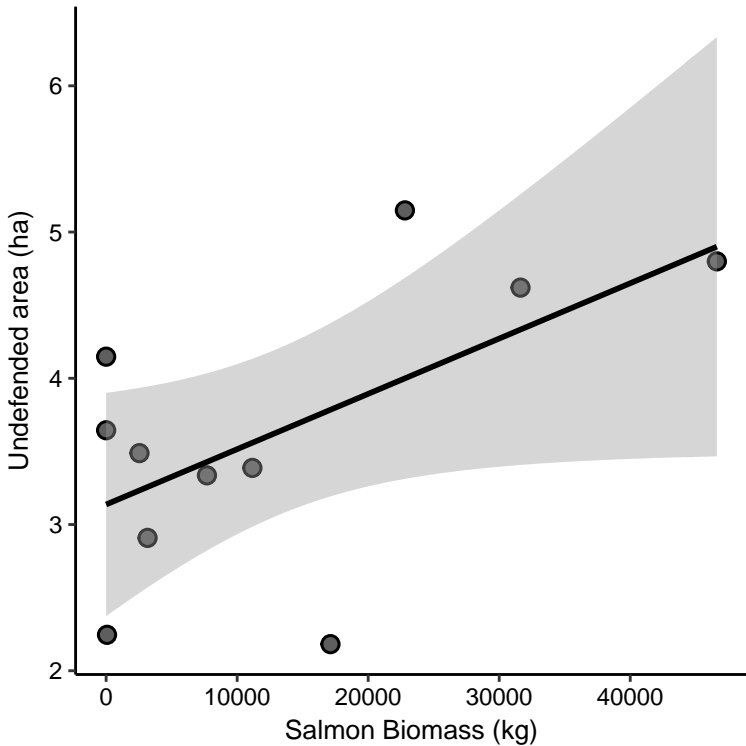

Supplement: S4 Fig — The dark line represents the best-fit line and the band represents the 95% confidence interval. (PDF) [file pone.0254314.s004.pdf]
