# Supplementary material for: Salmon subsidies predict territory size and habitat selection of an avian insectivore
Source: PLoS One. 2021 Jul 8;16(7):e0254314. doi: 10.1371/journal.pone.0254314 (PMC8266124; doi:10.1371/journal.pone.0254314)
Supplement: S1 Table — (PDF) [file pone.0254314.s005.pdf]

S1 Table Stream characteristics and salmon population data (2012-2015) for watersheds (n=11) in this study.

Stream	UTM Easting	UTM Northing	Catchment Area (km^2)	Spawning length (m)	Bankfull width	Mean No. Pink	Mean No. Chum	No. Territories	No. males banded
Ada	565113	5767615	9.8	435	11.1	155	2145	3	2
Beales	569502	5782472	6.5	300	10.9	1291	258	4	2
Bullock	562736	5806247	3.3	622	10.9	2524	4026	7	5
Clatse	579203	5798981	24.3	900	22.8	27500	3888	7	2
Fancy Right	567421	5767983	9.9	298	4.8	69	703	2	2
Fannie Left	563991	5766215	16.4	1500	12.8	8068	6269	7	4
Hooknose	579609	5775529	14.8	1800	16.9	3791	1889	4	4
Jane	564455	5767042	1.3	500	4.6	0	0	2	2
Kunsoot	560151	5777915	4.9	1500	13.1	13173	1999	3	2
Ripley	575560	5809303	15.7	0	14.7	0	0	2	2
Troupe	566176	5793788	1.6	332	4.4	10	4	3	1
