# Supplementary material for: Salmon subsidies predict territory size and habitat selection of an avian insectivore
Source: PLoS One. 2021 Jul 8;16(7):e0254314. doi: 10.1371/journal.pone.0254314 (PMC8266124; doi:10.1371/journal.pone.0254314)
Supplement: S2 Table — Habitat parameters tested include: Salmon = summed chum and pink salmon biomass, conifer cover = percent cover of all coniferous trees, riparian shrub cover = percent cover of riparian shrubs predominantly stink currant and salmonberry, male density = absolute male wren density per hectare, territory size = male wren territory size in hectares. Year (fixed effect) and stream (random effect) were included as variables in all models but are excluded from the table for clarity. K = number of parameters in model, ΔAICc = difference between the model AICc and the top model AICc, wi = model AICc weight, ER = evidence ratio. (PDF) [file pone.0254314.s006.pdf]

S4 Table AICc model selection analysis of linear regressions (full candidate model sets) describing Pacific Wren territory size, countersinging rate, and body condition response variables predicted by salmon biomass, wren density and environmental metrics. Habitat parameters tested include: salmon = summed chum and pink salmon biomass, conifer cover = percent cover of all coniferous trees, riparian shrub cover= percent cover of riparian shrubs predominantly stink currant and salmonberry, male density = absolute male wren density per hectare, territory size = male wren territory size in hectares. Year (fixed effect) and stream (random effect) were included as variables in all models but are excluded from the table for clarity. K = number of parameters in model, ∆AICc = difference between the model AICc and the top model AICc, w_i = model AICc weight, ER = evidence ratio.

response	parameters	K	∆AICc	w_i	ER
Territory size	salmon	3	0	0.51	1
	salmon + conifer cover	4	2.2	0.17	2.98
	null	2	3.0	0.12	4.39
	salmon + riparian shrub cover	4	3.4	0.09	5.51
	salmon + riparian shrub cover + conifer cover	5	4.7	0.05	10.64
	riparian shrub cover	3	5.8	0.03	18.07
	riparian shrub cover + conifer cover	4	8.9	0.01	86.05
proportional countersinging rate	male density	3	0	0.63	0.71
	salmon	3	2.4	0.19	0.71
	male density + salmon	4	3.7	0.10	0.71
	null	2	3.9	0.09	0.73
body condition	null	2	0	0.48	1
	relative territory size	3	0.4	0.41	1.17
	territory size	3	2.9	0.11	4.36
