# Supplementary material for: Salmon subsidies predict territory size and habitat selection of an avian insectivore
Source: PLoS One. 2021 Jul 8;16(7):e0254314. doi: 10.1371/journal.pone.0254314 (PMC8266124; doi:10.1371/journal.pone.0254314)
Supplement: S3 Table — Habitat parameters tested include: Salmon = summed chum and pink salmon biomass per stream (kg), high-nutrient shrubs = percent salmonberry and stink currant shrub cover, other shrubs = percent cover of shrubs blueberry, false azalea, and salal, conifer cover = percent cover of small conifer trees, shrub diversity = Shannon diversity index of all shrub species, hemlock = western hemlock stand basal area (m2 ha-1), redcedar = western redcedar stand basal area (m2 ha-1), alder = red alder stand basal area (m2 ha-1), spruce = Sitka spruce stand basal area (m2 ha-1), tree diversity = Shannon diversity index of all tree species, logs and stumps = percent cover of all large woody debris. Year (fixed effect) and stream (random effect) were included as variables in all models but are excluded in the table for clarity. K = number of parameters in model, ΔAIC = difference between the model AIC and the top model AIC, wi = model AIC weights, ER = evidence ratio. (PDF) [file pone.0254314.s007.pdf]

S5 Table All candidate models from the logistic regression models for probability of habitat selection by territorial male wrens. Habitat parameters tested include: salmon = summed chum and pink salmon biomass per stream (kg), high-nutrient shrubs = percent salmonberry and stink currant shrub cover, other shrubs = percent cover of shrubs blueberry, false azalea , and salal, conifer cover = percent cover of small conifer trees, shrub diversity = Shannon diversity index of all shrub species, hemlock = western hemlock stand basal area (m^2 ha^{-1}), redcedar = western redcedar stand basal area (m^2 ha^{-1}), alder = red alder stand basal area (m^2 ha^{-1}), spruce = Sitka spruce stand basal area (m^2 ha^{-1}), tree diversity = Shannon diversity index of all tree species, logs and stumps = percent cover of all large woody debris. Year (fixed effect) and stream (random effect) were included as variables in all models but are excluded in the table for clarity. K = number of parameters in model, ∆AIC = difference between the model AIC and the top model AIC, w_i = model AIC weights, ER = evidence ratio.

**response**	**hypothesis**	**parameters**	**K**	**∆AIC**	**w_i**	**ER**
Probability of habitat use	salmon & habitat interactions	salmon * redcedar + high-nutrient shrub + hemlock	7	0	0.607	1
	salmon & habitat interactions	salmon * redcedar + high-nutrient shrub + other shrub + hemlock	8	2.0	0.2266	2.68
	salmon & habitat	salmon + redcedar + high-nutrient shrub + other shrub + hemlock	7	3.8	0.0899	6.75
	salmon & habitat interactions	salmon * high-nutrient shrub + redcedar + other shrub + hemlock	8	5.8	0.0338	17.96
	habitat	redcedar + high-nutrient shrub + other shrub + hemlock	6	6.5	0.0241	25.19
	salmon & habitat	salmon + redcedar + high-nutrient shrub + other shrub + hemlock + alder + spruce	9	7.5	0.0143	42.45
	global model	salmon + redcedar + high-nutrient shrub + other shrub + hemlock + alder + spruce + conifer shrub + shrub diversity + tree diversity + woody debris	13	11.4	0.0021	289.05
	habitat	redcedar + hemlock + alder + spruce	6	11.9	0.0016	379.38
	habitat	redcedar + hemlock + alder + spruce + tree diversity	7	13.8	<0.001	>540
	habitat	high-nutrient shrub + other shrub + shrub diversity	5	24.2	<0.001	>540
	habitat	high-nutrient shrub	3	25.2	<0.001	>540
	salmon & habitat interactions	salmon * high-nutrient shrub	5	28.8	<0.001	>540
	habitat	woody debris	3	39.2	<0.001	>540
	salmon	salmon	3	39.6	<0.001	>540
